# Examining the Utility of Veterans Health Administration (VHA) Traumatic Brain Injury Screening among Women Veterans: Recommendations for Expansion to Include Interpersonal Violence

**DOI:** 10.3390/brainsci14080814

**Published:** 2024-08-14

**Authors:** Michelle M. Pebole, Katherine M. Iverson, Caitlin M. Bolduc, Julie M. Joyce, Caroline A. Sablone, Catherine B. Fortier

**Affiliations:** 1 The Translational Research Center for TBI and Stress Disorders (TRACTS), VA Boston Healthcare System, Jamaica Plain, Boston, MA 02130, USA; 2 Department of Psychiatry, Harvard Medical School, Boston, MA 02215, USA; 3 Women’s Health Sciences Division, National Center for PTSD, VA Boston Healthcare System, Boston, MA 02130, USA; 4 Department of Psychiatry, Chobanian & Avedisian School of Medicine, Boston University, Boston, MA 02118, USA

**Keywords:** women veterans, brain injury, screening, interpersonal violence, partner violence

## Abstract

Women veterans (WVs) are more likely than men veterans to experience traumatic brain injury (TBI) from causes unrelated to deployment. Yet, current Veterans Health Administration (VHA) TBI screening focuses on deployment. This study examines the utility of the VHA TBI screening tool for WVs. Using the Boston Assessment for TBI-Lifetime (BAT-L) as the gold standard, sensitivity and specificity of the VHA screen were identified for deployment and non-deployment injuries. Injuries missed by the screen were thematically described. Sensitivity and specificity were compared by context (research, clinical). Ninety WVs were included; fifty-three (60.9%) met TBI criteria per the BAT-L. For TBIs occurring during deployment, sensitivity was higher in research (89.1%) compared to clinics (61.7%); specificity was lower in research (60.7%) compared to clinics (93.0%). The BAT-L identified 27 non-deployment TBIs not captured by the VHA screen, most frequently from physical assault or sports. The VHA screen does not include non-deployment events; thus, non-deployment sensitivity and specificity could not be calculated. For lifetime TBIs (deployment + non-deployment etiologies), sensitivity was higher in research (73.5%) compared to clinics (48.9%). Specificity was lower in research (60.0%) compared to clinics (100.0%). Findings can inform improvements to TBI screening among WVs, including expansion for interpersonal violence.

## 1. Introduction

Women veterans (WVs) are the fastest-growing subpopulation of veterans, with rapidly increasing enrollment in healthcare through the Veterans Health Administration (VHA) among WVs [1,2]. The percentage of WVs is projected to increase from 6% to approximately 16% of the total veteran population in 2040, due in part to the large number of women involved in post-9/11 conflicts [1,3]. Although historically the military and VHA have focused primarily on enrolling and caring for male veterans [4,5], substantial increasing attention has been paid to the healthcare needs of the growing WV population. The VHA has made concerted efforts to increase attention to sex and gender disparities in quality of care [6,7]. These efforts include acknowledging the need for gender-sensitive services and measures that acknowledge the unique experiences of WVs. For example, in response to research demonstrating that WVs are at disproportionate risk for experiencing military sexual trauma and intimate partner violence, VHA implemented universal screening protocols for these types of violence [8,9]. Further examples of expansion of services relevant to WVs include implementation of IPV support programs [10] and establishing women’s health service delivery requirements [11]. Although progress has been made in providing options for gender-sensitive healthcare programs and services, there are still many opportunities to improve WVs’ health experiences from a trauma-informed lens [12].

Traumatic brain injury (TBI), defined as a physiological disruption in brain functioning resulting from an external force to the head, is a hallmark injury among post-9/11 veterans. Current reports estimate that between 11 and 15% of post-9/11 WV VHA patients screen positive for TBI with the VHA screener [13,14,15]. TBI is associated with detriments in clinically relevant domains such as cognitive difficulties, increased mental health diagnoses, functional disability, increased risk for cardiometabolic disease, and other medical comorbidities like chronic pain and poor sleep [16,17,18]. Critically, experiences of TBI differ between women and men veterans. WVs are more likely to experience TBI secondary to partner and sexual violence during and post military service and more likely to experience TBIs from etiologies unrelated to their deployment, such as motor vehicle accidents and falls, when compared to their men counterparts [19,20]. Further, research examining TBI-related outcomes among WVs has found that WVs with a history of TBI report worse psychosocial outcomes than their men counterparts. These include more neurobehavioral symptoms, headaches, and increased depression, as well as increases in concurrent mental and physical health symptoms when compared to male veterans [21,22,23,24,25]. These associations with clinically relevant and multimorbid health conditions underscore the importance of effectively identifying TBIs among post-9/11 WVs.

Since 2007, the VHA has screened all veterans who returned from deployment in post-9/11 conflicts for TBI [26]. The VHA TBI screening tool was designed to identify people at risk for having sustained a TBI during deployment by prompting the veteran about injuries sustained during deployment and the presence/persistence of resulting neurobiological symptoms [27]. This screening method was designed to be more sensitive than specific to align with its overarching goal of identifying veterans at risk for deployment-related TBI who had not been previously identified. Previous work indicates this screening tool has indeed produced moderate sensitivity for identifying deployment TBIs [28,29,30]. Yet, the focus of this screening tool on deployment TBIs limits its applicability for WVs, given that they often experience TBIs from different causes as compared to men veterans (including TBIs not related to deployment), and as such, is a notable limitation of this tool. For example, the VHA TBI screener (full text available in Supplementary Table S1) prompts veterans to identify injuries such as blasts or blows to the head but does not explicitly mention experiences of violence such as military sexual trauma. Moreover, these prompts are limited to experiences that occur during deployment and do not acknowledge common non-deployment injuries. This screening method thus systemically omits experiences that are highly relevant for WVs, decreasing the utility of the screening and missing an opportunity to provide WVs with gender-sensitive and tailored care. These are critical omissions, given the increasing number of WVs enrolling in VHA care who experience disproportionate risk for interpersonal violence and gender disparities in TBI outcomes, particularly given the clinical relevance of these injuries to physical and mental health outcomes.

In addition to these limitations, the setting of VHA TBI screen administration can impact the utility of the tool. The VHA TBI screening tool was meant to be administered by any clinical intake personnel. This means that veterans can receive TBI screening in a variety of clinical contexts that are otherwise unrelated to TBI services (e.g., dental or eye appointment). If a veteran screens positive, they are offered a referral to VHA polytrauma clinics, where they can receive comprehensive TBI assessments and evaluations to determine the presence or absence of military TBI. The diversity of clinical screening contexts means that a range of providers screen for TBI in contexts unrelated to brain health, which can negatively impact the accuracy of this screening method. This contrasts with TBI screens performed during clinical visits related to brain health (e.g., neurology or polytrauma clinics), where TBI-related knowledge and focus on brain and cognition-related symptomology during the visit often improves the sensitivity of TBI screening tools when compared to clinical contexts [31]. Similarly, TBI screens completed in the context of a TBI-related research study have been better able to identify TBIs compared to clinical settings [31]. However, given that the sex- and gender-specific utility of the TBI screen has been previously unexamined, it is currently unclear if this difference in screening utility between clinical and TBI research-focused contexts is consistent for TBI screening among WVs.

This study aims to address these research gaps regarding the utility of the VHA screening tool and to effectively identify injuries relevant for WVs. The primary aim is to explore the sensitivity and specificity of the VHA clinical screening measure for deployment and non-deployment etiologies and for injuries occurring across the lifespan (deployment + non deployment) among WVs. For these analyses, the gold-standard Boston Assessment of Traumatic Brain Injury-Lifetime (BAT-L) was used to identify TBIs occurring over the lifespan (deployment + non-deployment TBIs). To further explore gaps in screening, we report injury types among WVs identified on the BAT-L that were missed by current VHA screening methods. A secondary aim is to assess if the context of TBI screening (i.e., research vs. clinical) impacts the effectiveness of screens.

## 2. Materials and Methods

### 2.1. Participants

This study is a secondary analysis of WVs enrolled in a longitudinal cohort study at the Translational Research Center for TBI and Stress Disorders (TRACTS), a VA National Network Center for TBI, located at Boston and Houston VA Healthcare Systems. The TRACTS longitudinal cohort study began in 2009 and assesses the biological, psychological, cognitive, and neural functioning of post-9/11 veterans of the OIF/OEF/OND conflicts. Inclusion criteria are age between 18 and 75, military deployment in OEF/OIF/OND or other post-9/11 conflicts, or scheduled deployment. Exclusion criteria are history of neurological illness other than TBI; history of seizures (unrelated to TBI); current diagnosis of schizophrenia spectrum or other psychotic disorders or bipolar or related disorders; current suicidal and/or homicidal ideation with intent or plan; or cognitive disorder due to general medical condition other than TBI. In-depth information about TRACTS is available in McGlinchey et al., 2017 [32]. All veterans who completed baseline (Time 1) appointments and self-identified their sex assigned at birth as female were included in this analysis. All procedures were approved by the Boston Veterans Affairs Healthcare System Institutional Review Board and all participants provided informed consent.

### 2.2. Measures

**Assessment of PTSD symptoms:** As a part of the TRACTS study, all WVs included in this analysis were assessed for current and lifetime PTSD symptoms. PTSD status was assessed with the gold-standard clinician-administered interview, The Clinician Administered PTSD Scale (CAPS-IV) [33], which determined the presence of PTSD and its symptom severity. Diagnostic and Statistical Manual of Mental Disorders IV (DSM ’IV) criteria were used, given these were the criteria when the TRACTS longitudinal cohort study began enrollment (2010). Current and lifetime history of PTSD are reported in Table 1.

**VA TBI screen administered by research staff:** The VHA TBI screen was administered to all veterans in our sample by a member of the TRACTS research staff during the same evaluation in which the BAT-L was administered. Notably, the TBI screen was administered prior to the BAT-L clinical interview so that the BAT-L did not first cue them to injuries. The brief measure (included in full as Supplementary Table S1) includes 4 questions regarding exposure to a potential TBI event, loss or alteration of consciousness, and post-concussive symptoms at the time of the event and at the time of evaluation. A positive screen was indicated by yes on all four screening items, consistent with the clinical screen.

**VA TBI screen assessed by clinical screen:** VHA TBI screens were also obtained from WVs’ VA medical records. A Research Assistant reviewed the problem list, international classification of disease code (ICD) screen, polytrauma clinic, and general clinic notes for a clinician-administered TBI screen completed as a part of clinical care. Note that the clinician-administered VHA TBI screen may have been administered at various time points, including days, months, or even years apart from the BAT-L and research-administered VA TBI screen. A positive screen indicates yes responses on all four items.

**The Boston Assessment of TBI-Lifetime:** The Boston Assessment of TBI-Lifetime (BAT-L), a validated, semi-structured clinical interview with strong inter-rater reliability (>0.80), was used as the gold standard to identify and document TBIs [34]. The BAT-L has demonstrated strong correspondence with The Ohio State University Traumatic Brain Injury (TBI) Identification Method (OSU-TBI-ID; Cohen κ = 0.89; Kendall τ-b = 0.95 [34]). For this analysis, a positive history of lifetime TBI indicates that the participant sustained at least one head injury that meets the minimum criteria for mild TBI defined by the American Congress of Rehabilitation Medicine [35]. TBI assessments were conducted by doctoral- or master’s-level clinicians and reviewed at weekly diagnostic consensus meetings consisting of at least three doctoral-level psychologists.

### 2.3. Data Analysis

Analyses were completed with the Statistical Package for the Social Sciences (SPSS; version 20). All WVs enrolled in the longitudinal cohort study at the Translational Research Center for TBI and Stress Disorders and who identified their sex assigned at birth as female were included in this analysis. Since this study is a secondary exploratory analysis of the TRACTS cohort study, no power analysis was completed. Descriptive statistics for demographics, clinical characteristics, and type of sustained injuries are reported in Table 1. Associations between sociodemographic variables (age, race, education, PTSD, MST) and a history of lifetime TBI per the BAT-L were run to understand which factors were associated with experiencing lifetime TBI. All comparisons reported below reference the VHA TBI screen (administered in a research or clinical setting) relative to the BAT-L clinical interview as the criterion standard. The sensitivity and specificity of each screening tool were calculated to assess diagnostic accuracy. Sensitivity and specificity were calculated according to their respective formulas. Sensitivity identified the number of true positives, which was divided by the sum of true positives and false negatives. Specificity divided the number of true negatives by the sum of true negatives and false positives. The resulting sensitivity and specificity rates provide critical insight into the diagnostic performance of TBI screening in clinical and research settings.

## 3. Results

**Participants:** The final sample included 90 WVs with baseline data. Table 1 displays demographic characteristics and descriptive information for key study variables. In sum, just over half of this sample was White (57.8%), with an average age of 35.9 ± 9.7 years. Most had received either a Bachelor’s or advanced degree (88.8%) and completed their military service with the Army (65.6%). Approximately half (53.3%) met the diagnostic criteria for PTSD and 58.9% experienced a lifetime TBI per the gold-standard BAT-L clinical interview. Only a lifetime history of PTSD was significantly associated with TBI experienced over the lifetime (*p* = 0.047).

**Women veterans without a clinical TBI screen:** The first notable result was that almost 15% (*n* = 13; 14.4%) of WVs in this sample did not have a record of a clinical TBI screen in their electronic health record. These women were thus not included in clinical sensitivity/specificity analyses.

**Sensitivity and specificity of deployment, non-deployment, and lifetime TBIs for VHA TBI screen administered in clinical contexts:** Complete information on sensitivity and specificity for TBI type and context can be found in Table 2. For screens completed in a clinical context, sensitivity was highest (61.7%) and specificity was lowest (93.0%) for deployment TBIs. The clinical screen was not designed to detect any non-deployment TBIs and thus did not identify any. As such, sensitivity and specificity could not be calculated. For any lifetime TBIs (including both deployment and non-deployment injuries), sensitivity was worse (48.9%), and specificity was better (100%) than deployment sensitivity.

**Sensitivity and specificity of deployment, non-deployment, and lifetime TBIs for VHA TBI screen administered in research contexts:** Overall, screening performed in the context of TBI-focused research outperformed screening conducted in clinical settings. Sensitivity was highest for deployment injuries (89.1%) and was slightly lower for lifetime injuries (deployment + non-deployment injuries; 73.5%). Specificity was similar between deployment (60.7%) and lifetime (60.0%) injuries. Given that VHA TBI screens administered in research settings also could not assess non-deployment TBIs, sensitivity and specificity could not be calculated for this analysis.

**Non-deployment TBI etiologies currently missed by the VHA screening tool in both clinical and research settings:** The two most frequent non-deployment TBI etiologies that were not captured by VHA TBI screens were physical assaults (*n* = 14 TBIs) and sports-related injuries (*n* = 13 TBIs). Of the fourteen physical assaults captured by the BAT-L, ten were caused by assault by a partner, two were caused by an assault from a parental figure, and two were assault by a stranger. Six TBIs were reported from motor vehicle accidents, five from falls, and two from other accidents (i.e., wrestling with sibling, hit head on ceiling). 

## 4. Discussion

This study helps fill a significant research gap to better understand the utility of VHA TBI screening among WVs. A critical characteristic of this sample is that just under one-third experienced a non-deployment TBI per the gold-standard BAT-L that was not captured by the VHA TBI screen, regardless of the setting in which it was administered (i.e., research or clinical). This is consistent with research previously identifying that WVs experience high rates of TBI across the lifespan, often secondary to interpersonal violence [8,36]. Indeed, the injuries not captured by screening tools in this analysis most frequently resulted from physical assaults from partners, strangers, or caregivers. This points to a critical gap in VHA TBI screening methods for etiologies most relevant for WVs (e.g., physical assaults) across the lifespan. Identifying effective screening methods that capture relevant injury etiologies for WVs is critical, given that WVs experience disproportionate risk for TBI from intimate partner violence, childhood sexual assault, and military sexual trauma, often resulting in clinically relevant detriments to physical and mental health [37,38,39,40]. Screening for lifetime TBIs, including those caused by interpersonal violence and other non-deployment etiologies like accidents and falls, can enhance care for WVs by effectively identifying, validating, and addressing WVs’ unique experiences and needs from a trauma-informed lens. Further, screening for lifetime experiences of TBI among WVs would align with the recent shift within VHA towards expanding services offered for veterans experiencing partner and sexual violence over the lifetime [41,42], rather than exclusively during service (as has been the case for military sexual trauma, which by definition occurs during military service).

Another notable finding was that ~15% of this sample did not have a TBI screen documented in their clinical health record. This suggests that WVs may not be consistently screened for TBIs, despite the universal screening mandated for post-9/11 veterans, and may therefore miss the identification of potential TBIs. This is consistent with research finding that WVs may be screened for TBI at lower rates than men and that WVs are three times less likely than men veterans to receive comprehensive TBI assessments after a positive TBI screen [43]. Further, for all injuries (deployment and lifetime), when the VHA TBI screen was administered in a TBI research setting, it was better able to identify TBIs experienced by WVs than when administered in a clinical setting, resulting in higher sensitivity. This finding is consistent with previous TRACTS research documenting that when the VHA TBI screen was conducted in a research setting focused on TBI, a greater number of TBIs were correctly identified than when administered in clinical settings [31]. This finding is also consistent with studies examining screening for violent experiences relevant for WVs, such as intimate partner violence and military sexual trauma, which have consistently found clinical screening often underestimates the prevalence of violence when compared to research [44,45,46]. Together, these results suggest that VHA clinical screening likely underestimates the actual prevalence of TBIs among WVs. As such, liberal referrals to polytrauma assessment clinics, WV-specific health clinics (which may be more likely to validate WVs’ experiences address relevant injuries and concerns), or TBI-focused research may be warranted to ensure effective identification of injuries and treatment options for TBI-related health conditions among WVs.

Sensitivity was highest for TBIs occurring during deployment, was reduced for lifetime TBIs, and could not be calculated for non-deployment TBIs. High sensitivity for deployment TBIs was expected, given that the screen directly prompts for deployment related injuries. The lack of prompts for non-deployment injuries was the contributing factor for reducing the sensitivity for lifetime TBIs and the reason why sensitivity and specificity for non-deployment TBIs could not be completed. These findings suggest there is an opportunity to improve screening utility by capturing lifetime injuries most relevant for WVs. That the majority of TBIs from physical assaults in this sample were caused by intimate partners further highlights the importance of continued attention to the rapidly growing literature on IPV-related brain injury [37,47,48,49,50,51,52,53]. Clinical interviews such as the BAT-L and BATL/IPV [34,54] that cue veterans to potential injury contexts across the lifespan, including interpersonal violence, could be used to inform inclusive screening tools tailored to the unique needs of WVs. Early identification of TBI across deployment and non-deployment etiologies among WVs is especially relevant, given that WVs experience worse TBI-related psychosocial outcomes when compared to their men counterparts [19,21,40,55]. Further, detriments in physical and mental health may persist chronically, contributing to heightened risk for suicidality and long-term functional disability for WVs [50,56,57]. Improvements in TBI screening methods for WVs therefore present a significant opportunity to improve early identification and treatment of chronic health conditions often associated with TBI.

## 5. Limitations

This study was retrospective and cross-sectional, thus limiting the analysis of injuries relating to TBI assessments. Moreover, the accuracy of screening methods may have been impacted by self-report biases. However, our gold-standard BAT-L method was a clinically administered interview with rigorous methods, including clinical consensus team agreement for all injuries. The sample size was modest for this secondary analysis. Future work with larger samples of WVs and civilian women is needed and could also address intersectional issues in TBI (i.e., race/ethnicity, age, socioeconomic status), which would be informative for the field. Lastly, selection bias and other barriers to engaging in research (e.g., lack of time or transportation, unwillingness to travel to the VA Hospital) may impact the generalizability of this analysis to the broader population of WVs. Although interpersonal violence, including IPV and sexual violence, tends to be more common among WVs relative to their civilian counterparts [9,58,59], the prevalence of these types of violent exposures among women in the general population cannot be understated. Thus, there remains a need to understand the utility of other screening tools for detecting TBIs among all women.

## 6. Conclusions

This study addressed several significant gaps in identifying the accuracy of TBI screening among WVs, which has implications for understanding the prevalence and unique experiences of TBI among WVs. Our findings present several recommendations for TBI screening among WVs. First, screening for lifetime TBIs from a variety of etiologies (e.g., partner violence and military sexual trauma, accidents) instead of exclusively focusing on deployment-related TBIs would benefit WVs, given their high burden of TBIs occurring over the lifespan from diverse etiologies that are distinct from those experienced by men. Expanding current screening tools to injuries most prevalent among WVs would provide WVs with more holistic care which identifies and validates their unique experiences. Additionally, the under-identification of TBIs in clinical care suggests that more liberal screening and referrals for comprehensive TBI assessments in polytrauma clinics, WV-specific care clinics which validate the experiences of WVs, or research settings may improve care for WVs. Improving the effectiveness of TBI screening tools for WVs is critical, given the many health disparities WVs face in physical and mental health because of these injuries.

## Figures and Tables

**Table 1:** Sociodemographic information for the sample.

Full Sample (N = 90)	N(%)/Mean ± SD
Age	35.9 ± 9.73
Race	
White	52(57.8)
Black	23 (25.6)
Other/Unknown	15 (16.6)
Hispanic/Latina	19 (21.1)
Education	
High School/GED	10 (11.1)
Bachelor’s Degree	61 (67.7)
Graduate or higher	19 (21.1)
Service Branch	
Army	59 (65.6)
Navy	8 (8.9)
Airforce	20 (22.8)
Marines	4(4.4)
Lifetime PTSD	77 (85.6)
Current PTSD	48 (53.3)
Lifetime TBI (BAT-L)	53 (58.9)
Lifetime TBI (Clinical)	24 (26.7)
MST screen+	44 (48.9)

**Note:** SD: standard deviation; MST+: screen positive for military sexual trauma (per screening in clinical care); PTSD: post-traumatic stress disorder (CAPS-IV); TBI: traumatic brain injury (BAT-L); GED: General Educational Development.

**Table 2:** Research and clinical positive TBI screens compared to gold-standard BAT-L positive TBI.

	BAT-L	Research TBI Screen		Clinical TBI Screen	
		Sensitivity	Specificity	Sensitivity	Specificity
Lifetime TBI	53	73.5%	60.0%	48.9%	100%
Deployment TBI	37	89.1%	60.7%	61.7%	93.0%
Non-Deployment TBI	27	-	-	-	-

## Data Availability

The datasets presented in this article are not readily available because the data are owned by the Boston Veteran Affairs Healthcare System and are subject to oversight by the VA.

## Supplementary Materials

The following supporting information can be downloaded at: https://www.mdpi.com/article/10.3390/brainsci14080814/s1. Supplemental Table S1. VHA TBI Screening Tool.
