# Supplementary material for: Examining the Utility of Veterans Health Administration (VHA) Traumatic Brain Injury Screening among Women Veterans: Recommendations for Expansion to Include Interpersonal Violence
Source: Brain Sci. 2024 Aug 14;14(8):814. doi: 10.3390/brainsci14080814 (PMC11352346; doi:10.3390/brainsci14080814)
Supplement: Supplementary file 1 [file brainsci-14-00814-s001.zip › brainsci-3127265-supplementary.pdf]

Supplemental Table S1. VHA TBI Screening Tool.

**1.** During any of your OIF/OEF deployment(s), did you experience any of the following events?	1 ☐ Blast or explosion (IED, RPG, Landmine, Grenade, etc)<br>2 ☐ Vehicular accident/crash (any vehicle including aircraft)<br>3 ☐ Fragment wound or bullet wound above the shoulders<br>4 ☐ Fall<br>5 ☐ Blow to head (head hit by falling/flying object, head hit by another person, head hit against something, etc)
**2.** Did you have any of these immediately afterwards?	1 ☐ Losing consciousness/“knocked out”<br>2 ☐ Being dazed, confused, or “seeing stars”<br>3 ☐ Not remembering the event<br>4 ☐ Concussion<br>5 ☐ Head injury
**3.** Did any of the following problems begin or get worse afterwards?	1 ☐ Memory problems or lapses<br>2 ☐ Balance problems or dizziness<br>3 ☐ Sensitivity to bright light<br>4 ☐ Irritability<br>5 ☐ Headaches<br>6 ☐ Sleep problems
**4.** In the past week, have you had any of the symptoms from Section 3?	1 ☐ Memory problems or lapses<br>2 ☐ Balance problems or dizziness<br>3 ☐ Sensitivity to bright light<br>4 ☐ Irritability<br>5 ☐ Headaches<br>6 ☐ Sleep problems
